# Aggregate Effects of Yoga-Based Volitional Breathing Interventions on Perceived Stress and Anxiety Levels Among Medical Students Worldwide: A Systematic Review and Meta-Analysis

**DOI:** 10.7759/cureus.109256

**Published:** 2026-05-20

**Authors:** Anupinder Thind, Pravin M Pisudde, Sonia Kochhar

**Affiliations:** 1 Department of Physiology, All India Institute of Medical Sciences, Bathinda, Bathinda, IND; 2 Department of Community and Family Medicine, All India Institute of Medical Sciences, Bathinda, Bathinda, IND

**Keywords:** anxiety, breathing exercises, medical students, mindfulness, stress, systematic review, yoga

## Abstract

Medical education has long been acknowledged as a very stressful career path that exposes students to greater chances of anxiety and burnout. The purpose of this systematic review and meta-analysis was to determine the overall impact of yoga-based volitional breathing (pranayama) interventions on self-perceived stress and anxiety among medical students worldwide. The review analysis was conducted following the Preferred Reporting Items for Systematic Reviews and Meta-Analyses (PRISMA) guidelines, and seven databases were searched from 2010 to 2025. Potential studies were limited to those that concerned interventions involving only pranayama. Seven studies were included, comprising one randomized controlled trial (RCT) and six single-arm studies. A random-effects model was used to compute standardized mean differences (SMD). The meta-analysis of the seven studies in general revealed a significant, large decrease in stress/anxiety (SMD = -1.25, 95% CI: -1.64 to -0.86, p < 0.001), but heterogeneity (I² = 67%) was high. Subgroup analysis indicated a significantly larger effect for short-term (≤8 weeks) programs (SMD = -1.68, 95% CI: -3.13 to -0.22) compared to long-term (>8 weeks) programs (SMD = -1.06, 95% CI: -1.46 to -0.65), with the between-group difference being statistically significant (Q = 9.71, p = 0.002). There were no notable differences in the main outcome measure or by mode of delivery. The effect size of the single RCT, which had a low risk of bias, was very large (SMD = -1.78). It was established that pranayama interventions had significant stress and anxiety reductions in medical students. Although additional high-quality RCTs are required, these results support the inclusion of a structured pranayama intervention in student wellness programs as a powerful and viable mental health promotion approach.

## Introduction and background

Medical education has long been recognized as a demanding professional trajectory due to its extended duration and considerable emotional burdens on students [1]. Contributing factors included high familial expectations, prolonged academic and clinical hours, a dense curriculum, and frequent sleep deprivation. These factors were reported to heighten susceptibility to stress, a condition that could escalate without appropriate intervention [2]. Consequently, a significant prevalence of psychological concerns, ranging from anxiety and stress to more severe psychiatric disorders, was documented within this population [3].

Volitional yoga breathing, defined as the conscious regulation of breath rate and depth to influence mental well-being and cognitive function, has been proposed as a potential non-pharmacological intervention [4]. These practices were typically categorized as high-frequency yoga breathing (HFYB) and low-frequency yoga breathing (LFYB). HFYB, exemplified by techniques such as Kapalabhati (skull-shining breath), involves rapid, forceful breaths at frequencies typically exceeding 60 breaths per minute [4-6]. Research has indicated that HFYB can significantly modulate brain activity, increasing alpha-wave production in temporal and frontal regions and gamma-wave activity in occipital, temporal, and frontal areas [5]. Furthermore, its influence on autonomic function, evidenced by a parasympathetic shift in heart rate variability (HRV), has been observed [6].

In contrast, LFYB involves slow, controlled breathing at frequencies typically ranging from one to six breaths per minute [7,8]. Nostril-regulated LFYB techniques, including right-nostril inspiration (Suryabhedana Pranayama) and left-nostril breathing (Chandrabhedana Pranayama), have been associated with improved task performance through enhanced focus and attention span [7], alongside respiration-mediated increases in parasympathetic activity [8]. Slow-paced pranayama (e.g., at six breaths per minute) has been found to increase baroreflex sensitivity and vagal activity [9]. Another LFYB practice, Brahmari Pranayama (bumblebee breath), has also been reported to elevate vagally mediated HRV [5].

Despite India's position as one of the world's largest medical education systems, contemporary health initiatives within the country frequently overlook stress and anxiety as emergent health concerns among medical trainees. Promoting mental well-being in this cohort was considered crucial, as it could lead to improved quality of life, encourage healthier lifestyle habits, reduce the risk of substance abuse, and help prevent chronic diseases. This systematic review and meta-analysis, therefore, aimed to assess the aggregate effect of yoga-based volitional breathing (pranayama) interventions on perceived stress and anxiety among medical students globally.

## Review

Methodology

The systematic review was conducted, and a meta-analysis was performed following the Preferred Reporting Items for Systematic Reviews and Meta-Analyses (PRISMA) guidelines [10].

*Development* *and* *Execution* *of* *Search* *Strategy*

A comprehensive search strategy was developed to locate all relevant literature. The strategy was designed by integrating key concepts related to the target population (medical students), the intervention (pranayama), and the outcome of interest (anxiety, stress, cognition). Controlled-vocabulary terms (e.g., medical subject headings (MeSH) in PubMed) were used, and free-text keywords were combined with them to increase sensitivity. The last search query used Boolean operators (AND, OR) and syntax modifiers such as truncation of word variations and quotation marks for phrase searching. This main method was further adjusted to the indexing languages and field codes of each selected electronic database, such as PubMed, Scopus, Web of Science, PsycINFO, Cochrane CENTRAL, EMBASE, and IndMED. To ensure contemporary relevance, a publication date filter was used to include articles published between 2010 and 2025, and only articles written in English were considered (Table 1).

**Table 1:** Systematic search strategy across electronic databases MeSH: medical subject headings, EMTREE: Elsevier life science thesaurus

Search query components (Boolean operators)	Applied filters	Syntax/modifiers
Population: ("Students, Medical" OR "Medical Students" OR "medical trainee*") Intervention: ("Breathing Exercises" OR "Pranayama" OR "Yoga Breathing" OR "Volitional Breathing" OR "Kapalabhati" OR "Bhramari" OR "Alternate Nostril Breathing") Outcome: ("Anxiety" OR "Stress, Psychological" OR "mental well-being" OR "cognition" OR "attention")	Humans; Publication Date: From 2010 to Present; Language: English	PubMed: MeSH terms & text words; Truncation (*); Phrase searching (" ") Other DBs: Adapted field codes (e.g., TITLE-ABS-KEY in Scopus)

To supplement the electronic database search and reduce the risk of publication bias, the reference lists of each included study and all relevant systematic reviews were manually searched. During screening and data extraction, two reviewers used Rayyan (Rayyan Systems Inc., Cambridge, MA, US) to perform all processes [11]. Any misalignments or contradictions that emerged between the reviewers at any point, whether during study selection, data extraction, or risk-of-bias assessment, were first addressed by the two. If no consensus was reached, the conflict was escalated to a third senior reviewer, whose decision was final to ensure an unbiased and consistent result.

*Application* *of* *the* *PICO* *Framework* *for* *Study* *Selection*

The criteria for including studies in the meta-analysis were strictly based on the PICO (population, intervention, comparator, outcome) framework [12]. The target group was clearly stated and understood to be the undergraduate or graduate medical students from different healthcare disciplines. Only interventions meeting the criteria of being structured and based on yoga techniques for volitional breathing were included; those that combined pranayama with other yoga components were not considered because their effects could not be isolated. The relevant outcomes included both primary psychological outcomes (assessed through standardized measures of anxiety and stress) and secondary cognitive or autonomic measures. Only the quantitative research designs were included, including RCTs and controlled non-randomized studies; qualitative reports, descriptive studies, and protocols were filtered out (Table 2).

**Table 2:** Study eligibility criteria based on the PICO framework GAD-7: generalized anxiety disorder 7-item scale, HRV: heart rate variability, MBBS: bachelor of medicine, bachelor of surgery, MD: doctor of medicine, PICO: population, intervention, comparator, outcome (framework), PSS: perceived stress scale, RCTs: randomized controlled trials, STAI: state-trait anxiety inventory [12]

Category	Inclusion criteria	Exclusion criteria
Population (P)	Students enrolled in an undergraduate or graduate medical degree program from different healthcare disciplines (e.g., MBBS, nursing, dentistry), irrespective of year of study.	Practicing physicians or residents.
Intervention (I)	Structured pranayama (e.g., Kapalabhati, Bhramari, Nadi Shodhana, slow-paced pranayama) as the primary, stand-alone intervention. The practice could be supervised or unsupervised.	Multi-modal interventions where the effect of breathing exercises could not be isolated (e.g., yoga combining asana, pranayama, and meditation); non-yogic breathing techniques (e.g., Buteyko).
Comparator (C)	Active control (e.g., non-pranayama relaxation, physical exercise) or passive control (e.g., wait-list, no intervention, treatment as usual).	Not applicable
Outcomes (O)	Primary: Validated scales measuring self-reported anxiety (e.g., GAD-7, STAI) or perceived stress (e.g., PSS). Secondary: Objective or performance-based measures of cognition (e.g., attention, memory tests), HRV parameters.	Studies reporting only qualitative outcomes or physiological measures unrelated to the review's outcomes.
Study design	RCTs and non-randomized controlled intervention studies (e.g., quasi-experimental, pre-post).	Case reports, case series, narrative reviews, editorials, conference abstracts without full data, and protocols.

*Structured* *Data* *Extraction* *Procedure*

After the ultimate selection of studies, systematic data extraction was adapted to gather pertinent data. To achieve consistency and completeness, an electronic data extraction form was created in Microsoft Excel (Microsoft Corp., Redmond, WA, USA) as a standardized, pre-pilot version. Data were extracted in two reviews by researchers and then cross-checked to ensure the same data were obtained in both studies. The form included detailed information across several domains: (1) study identifiers and characteristics: author names, publication year, country, study design, and sample size; (2) participant details: medical school year, mean age, and gender ratio; (3) intervention details: type of volitional breathing practice, duration of session, frequency, duration of overall intervention, and information on instructor/supervision; (4) comparator group description; and (5) outcome data: means, standard deviations (SDs), and sample sizes of intervention and control groups at baseline and post-intervention. Missing or unclear data were requested from the respective authors.

*Assessment* *of* *Methodological* *Quality* *and* *Risk* *of* *Bias*

Two standardized tools were used to critically evaluate the methodological rigor and risk of bias in the individual studies. In randomized controlled trials (RCTs), the Cochrane Risk of Bias tool (RoB 2) was used [13], which assesses five domains of bias. For non-randomized studies, the ROBINS-I tool was used [14]. The ratings of each study were low, with some concerns and a high risk of bias. A funnel plot was created to evaluate the possibility of publication bias in the aggregate body of evidence, with the standard error of each study's effect estimate as the x-intercept and the effect size (e.g., standardized mean difference (SMD)) as the y-intercept. The funnel plot was tested for both visual and statistical asymmetry using Egger's linear regression test [15].

*Data* *Synthesis* *and* *Analytical* *Methods*

Statistical analyses were performed using RevMan version 5.4 (The Cochrane Collaboration, London, England, UK) [16] and Stata version 17 (StataCorp LLC, College Station, TX, US) [17]. For continuous outcome measures (anxiety and stress scores), the pooled effect size was estimated as an SMD with a 95% confidence interval (CI), since different measurement scales were likely used across studies. All meta-analyses were conducted a priori using a random-effects inverse-variance model to account for unforeseen clinical and methodological heterogeneity across studies. To test statistical heterogeneity, the I² statistic was used. In the absence of adequate data, pre-planned subgroup analyses were performed to investigate potential sources of heterogeneity by intervention period, primary outcome measure, and mode of delivery. The strength of the primary meta-analysis was tested through sensitivity analyses that sequentially eliminated studies rated as having an overall high risk of bias.

During data extraction, several outcome data formats were encountered and handled as follows. For studies reporting means and SDs at baseline and post-intervention, these values were extracted directly for meta-analysis. For studies reporting only medians with ranges or interquartile ranges, established statistical conversion methods were applied to estimate means and SDs where sufficient data were available. For studies reporting only median values without SDs, an SD of 6 was imputed based on the average SD from comparable studies using the same outcome measure (PSS) in similar populations. For studies reporting only p-values for outcome measures, an approximate effect size (Cohen's d = -1.04) was back-calculated from the t-statistic derived from the p-value and degrees of freedom, as described in the Cochrane Handbook. This approach was explicitly noted as an estimation, and the associated CIs reflected the resulting uncertainty.

For all single-arm pre-post studies included in the meta-analysis, the within-group standardized mean change was calculated using the formula: $d = \frac{M_{\mathrm{post}} - M_{\mathrm{baseline}}}{SD_{\mathrm{pooled}}}$, where

$SD_{\mathrm{pooled}} = \sqrt{\frac{SD_{\mathrm{baseline}}^{2} + SD_{\mathrm{post}}^{2}}{2}}$.

The variance of d was calculated as Var(d) = 2(1 - r)/n + d²/2n, assuming a conservative pre-post correlation of r = 0.5 in the absence of reported correlations. A sensitivity analysis was conducted using alternative correlation values (r = 0.3 and r = 0.7) to assess the robustness of the pooled effect to this assumption.

Results

*Study* *Selection* *Process*

**Figure 1:**
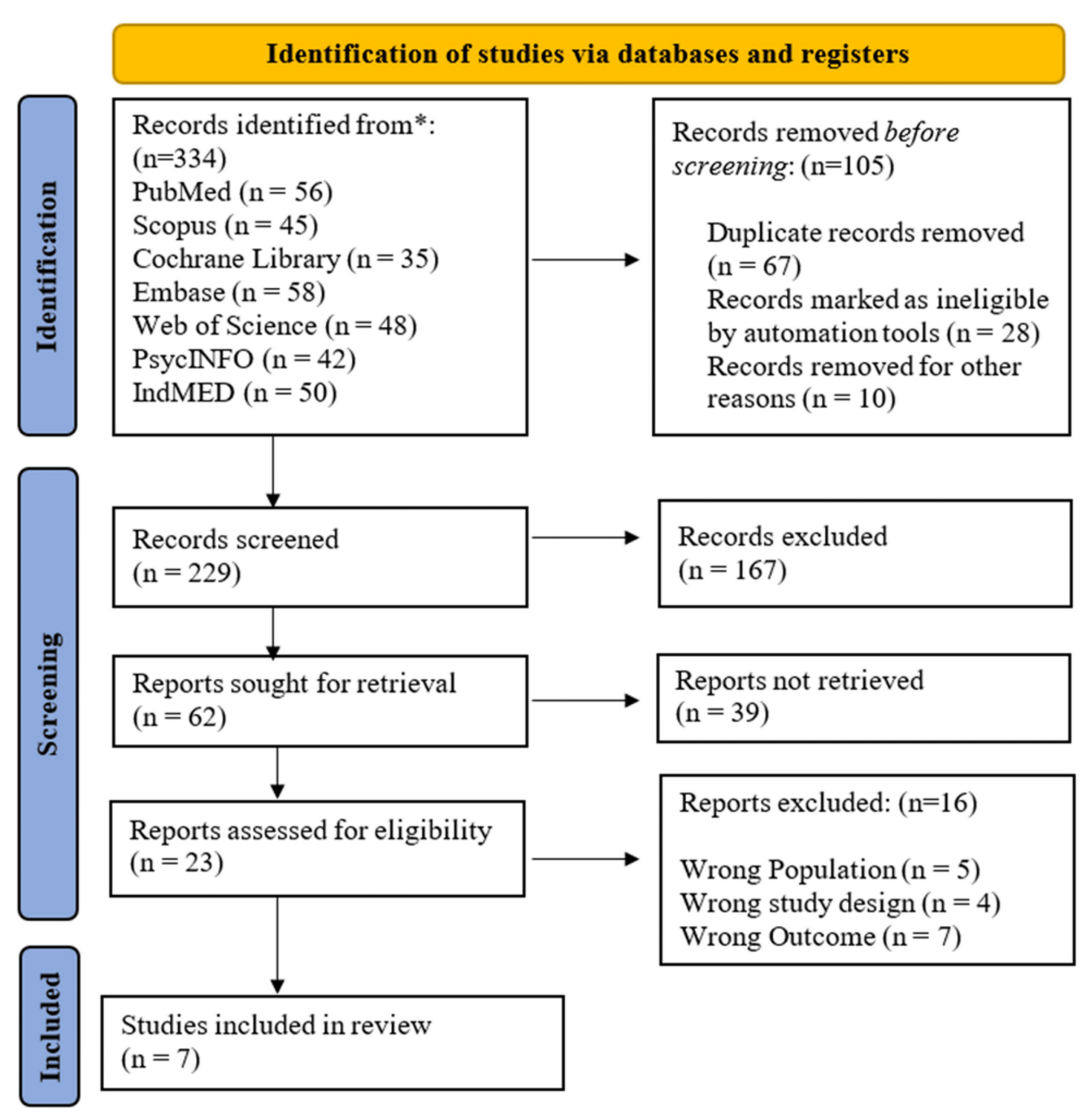
PRISMA flow diagram of the systematic review on pranayama interventions for medical students PRISMA: Preferred Reporting Items for Systematic Reviews and Meta-Analyses [10]

The initial search in seven large databases provided 334 records. After deleting 105 records, most of them because of duplication, 229 distinct records were subjected to title and abstract screening. This step eliminated 167 records, leaving 62 reports for full-text retrieval; 39 of them were not retrieved. As a result, 23 full-text papers were evaluated against the predefined eligibility criteria. Among them, 16 studies were excluded. Eventually, seven studies met all inclusion criteria and were included in the systematic review and meta-analysis (Figure 1) [18-24].

The properties of seven studies summarized in Table 3 tested the effects of pranayama interventions on the mental and physiological well-being of medical students. All studies were published between 2010 and 2025, and the majority were single-arm pre-post studies (n = 5), with one RCT and one quasi-experimental study with a comparison group.

**Table 3:** Demographic and methodological characteristics of included studies MBBS: bachelor of medicine, bachelor of surgery, MS: medical student, NR: not reported, RCT: randomized controlled trial

Study (author, year)	Country	Study design	Sample size (N)	Student year	Mean age (years)	Gender distribution
Gerbarg et al. (2023) [18]	Puerto Rico/USA	Pilot pre-post	12	MS1 and MS2	NR	7 female, 6 male
Muthe et al. (2025) [19]	India	Quasi-experimental	140 (50 cases, 90 comparisons)	First-year MBBS	Not specified	Not specified
Smitha (2016) [20]	India	Prospective pre-post	60	Mixed MBBS years	Not reported	Mixed (NR)
Manzoor et al. (2025) [21]	India	RCT	170 (85/group)	Not specified	~21.5	~44.7% M, ~55.3% F
Nagendrappa et al., (2025) [22]	India	Prospective single-group	60	Not specified	20.38 ± 0.87	23 Male, 37 Female
Sudhir et al. (2015) [23]	India	Observational pre-post	30	Various MBBS phases	19-25	12 Male, 18 Female
Dhanvijay et al. (2018) [24]	India	Pre-post observational	60	First-year medical	18-22 (range)	Not specified

The interventions primarily consisted of pranayama-based practices, alternating nostril breathing (Nadi Shodhana/Shuddhi) and slow/coherent breathing, with session lengths of five minutes (1) or 12-week programs (maximum). Validated psychological measures, including the PSS, BAI, and DASS-21, consistently demonstrated the primary results. However, several studies also reported autonomic variables such as heart rate variability (HRV) and blood pressure (Tables 4-5).

**Table 4:** Intervention and comparator characteristics of included studies ANB: alternate nostril breathing, BBM-IC: breath-body-mind – introductory course, NS: not specified

Study (author, year)	Intervention type	Session details	Total duration	Instructor/supervision	Comparator group
Gerbarg et al. (2023) [18]	BBM-IC	4h/day × 3 days + 45 min/week × 6 wks	9 weeks	Certified BBM teachers	None (single-arm)
Muthe et al. (2025) [19]	Structured yoga and meditation (incl. pranayama)	60 min/day, 5 days/week	6 weeks	Structured program (details NS)	Non-participant cohort (n = 90)
Smitha (2016) [20]	ANB	30 min/day, 5 days/week	3 months	Supervised practice	None (single-arm)
Manzoor et al. (2025) [21]	Structured yogic breathing (Nadi Shodhana, Kapalabhati, Bhastrika, Sheetal)	~30 min/session, 5 days/week	5 weeks	Qualified instructors, weekly supervision	Physical activity (matched duration/frequency)
Nagendrappa et al. (2025) [22]	Alternate nostril breathing (Nadi-Shodhana Pranayama)	15-20 min/day, 5 days/week	3 months	Likely instructor-guided initially	None (single-arm)
Sudhir et al. (2015) [23]	Alternate nostril breathing (Nadi Shodhana Pranayama)	20 min/day, daily	3 months	Supervised practice	None (single-arm)
Dhanvijay et al. (2018) [24]	Nadi Shuddhi Pranayama (ANB)	15 min daily, daily	12 weeks	Certified yoga trainer	None (single-arm)

**Table 5:** Outcome data from included studies BAI: Beck anxiety inventory, BPQ: body perception questionnaire, DASS-21: depression, anxiety and stress scale-21, DBP: diastolic blood pressure, EFI: exercise-induced feeling inventory, HRV: heart rate variability, IHG: isometric handgrip, PA: physical activity, PHQ-9: patient health questionnaire-9, PSQI: Pittsburgh sleep quality index, PSS: perceived stress scale, RMSSD: root mean square of successive differences, SBP: systolic blood pressure, SD: standard deviation, SDNN: standard deviation of NN intervals, SQS: sleep quality scale

Study (author, year)	Outcome domain	Specific measure	Baseline	Post-intervention	Significance/notes
Gerbarg et al. (2023) [18]	Psychological	EFI tranquility	-	↑	p = 0.005
		BPQ supradiaphragmatic reactivity	-	↓	p = 0.04
		SQS (sleep)	-	Trend ↑	p = 0.06
		PHQ-9 (depression)	-	Trend ↓	p = 0.078
Muthe et al. (2025) [19]	Psychological (case)	PSS-10 (stress)	24.6 ± 4.2	17.2 ± 3.8	p < 0.001
		BAI (anxiety)	23.1 ± 5.0	15.6 ± 4.2	p < 0.001
	Psychological (comparison)	PSS-10 (stress)	23.9 ± 4.5	23.2 ± 4.6	p > 0.05
		BAI (anxiety)	22.8 ± 5.3	22.1 ± 5.1	p > 0.05
Smitha (2016) [20]	Physiological	Heart rate	Decreased	-	p < 0.05
		DBP	Decreased	-	p < 0.05
		Galvanic skin resistance	Increased	-	p < 0.05
		HRV (SDNN, RMSSD)	Increased	-	p < 0.05
	Psychological	PSS (stress)	Decreased	-	p < 0.05
	Other	Auditory threshold (both ears)	Decreased	-	p < 0.05
Manzoor et al. (2025) [21]	Psychological	DASS-21 stress (yoga)	34.66 ± 6.21	20.23 ± 5.84	p < 0.001 vs. control
		DASS-21 stress (PA control)	28.71 (SD NR)	25.02 (SD NR)	-
		PSQI sleep (yoga)	4.77 ± 1.05	4.37 ± 0.98	p = 0.08 (within)
		PSQI sleep (PA control)	3.96 (SD NR)	3.00 (SD NR)	p = 0.012 (between, PA superior)
Nagendrappa et al. (2025) [22]	Psychological	PSS (stress)	21.62 ± 5.34	14.85 ± 4.60	p < 0.001
	Other	Auditory threshold (R)	7.51 ± 4.08 dB	5.17 ± 3.22 dB	p < 0.001
		Auditory threshold (L)	9.23 ± 7.48 dB	5.70 ± 3.82 dB	p < 0.001
Sudhir et al. (2015) [23]	Psychological	PSS (stress)	21.90 ± 6.08	16.37 ± 5.18	p < 0.001
	Other	Overall relaxation %	58.30 ± 11.46	62.90 ± 8.51	p < 0.005
Dhanvijay et al. (2018) [24]	Psychological	PSS (stress)	Median: 19	Median: 14	p < 0.001
	Physiological	Heart rate	76.88 bpm	72.67 bpm	p < 0.001
		SBP	114.2 mmHg	109.93 mmHg	p < 0.001
		DBP	75.60 mmHg	71.43 mmHg	p < 0.001
		DBP response to IHG	19.73 mmHg	16.67 mmHg	p < 0.001

*Comparative* *Risk*-*of*-*Bias* *Assessment*

**Figure 2:**
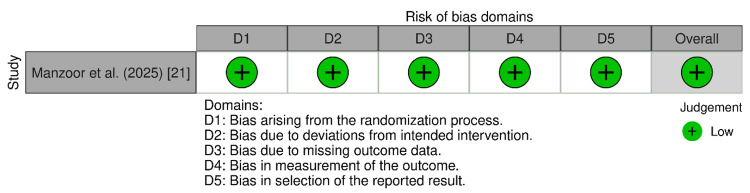
Graphical representation of risk of bias in RCTs using the Cochrane RoB 2 tool: domain-level and overall judgments RCT: randomized controlled trial [13]

**Figure 3:**
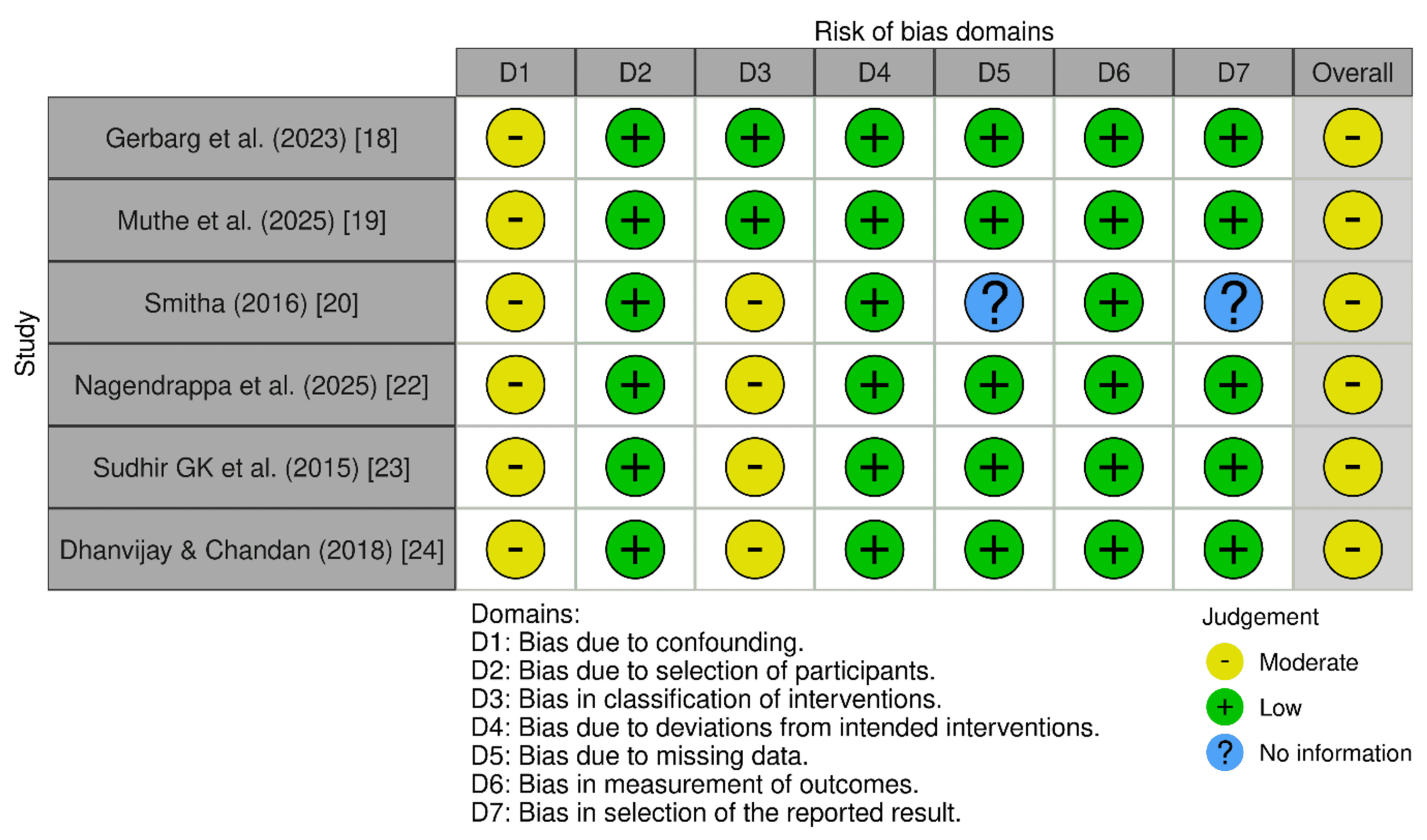
Graphical representation of risk of bias in non-randomized studies using the ROBINS-I tool: summary of judgments across seven domains [14]

Risk of bias: For the single RCT [21], the RoB 2 tool indicated low risk of bias across all domains, indicating high methodological rigor (Figure 2). On the other hand, the evaluation of the six non-randomized studies (mainly single-arm pre-post designs) using the ROBINS-I tool showed a moderate risk of bias across all studies. This was primarily driven by the inherent risk of confounding (domain 1), as no control group was mentioned (Figure 3).

**Figure 4:**
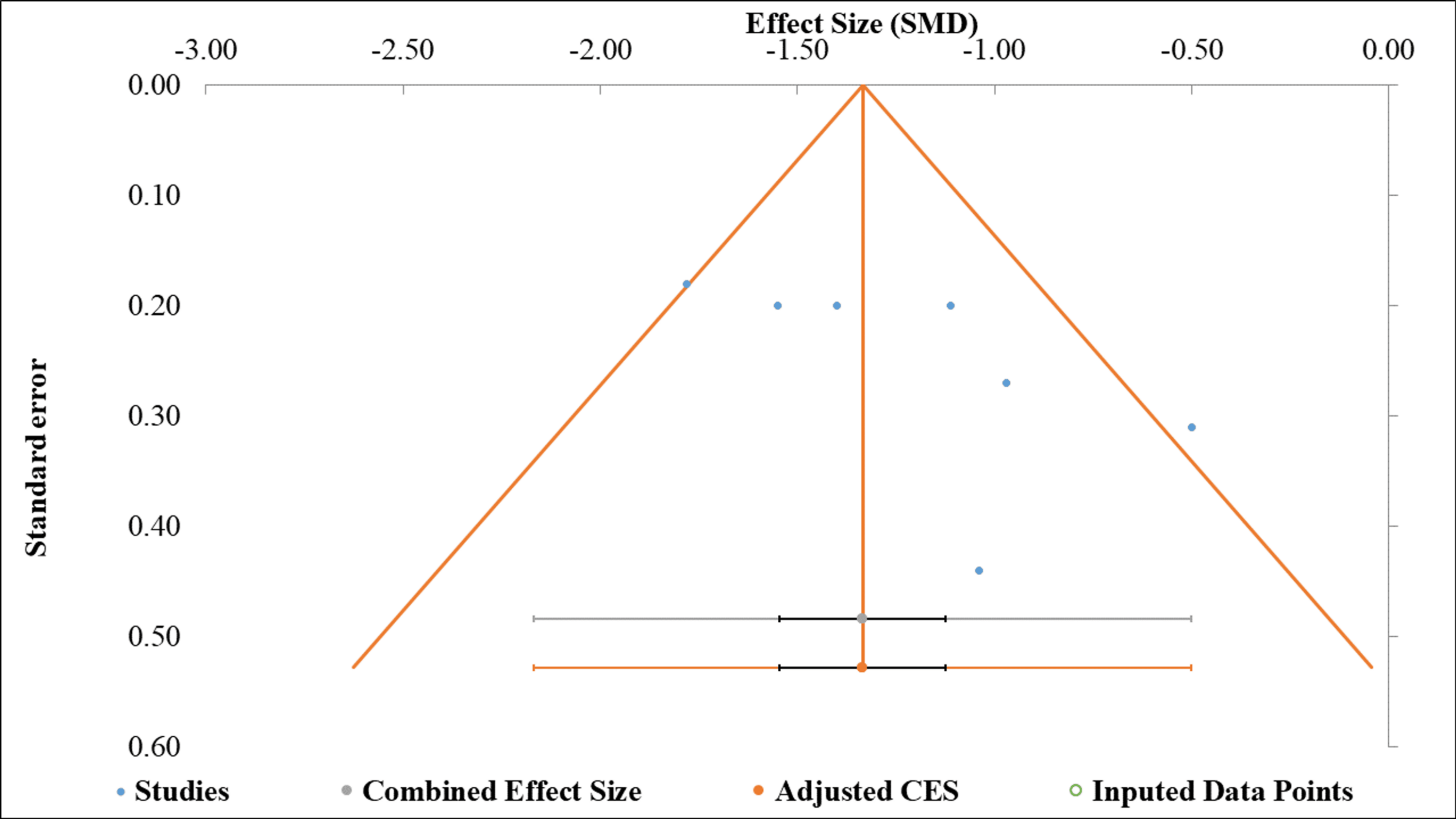
Assessment of publication bias using a funnel plot SMD: standardized mean difference, CES: combined effect size [15]

Publication bias: Figure 4 showed a slight skew, which could have led to an overrepresentation of small studies with null or opposite findings. A statistically non-significant intercept was obtained in the Egger linear regression test (p = 0.092). In contrast, the trend (negative slope = -2.29) suggested that slightly larger effect sizes may be found in smaller studies. Furthermore, it was acknowledged that with only seven included studies, the statistical power of Egger's test is limited, and the results should be interpreted with caution (Table 6) [15].

**Table 6:** Assessment of publication bias using Egger’s test CI: confidence interval [15]

Parameter	Estimate	Std. error	95% CI-lower limit	95% CI-upper limit
	4.34	2.08	-0.76	9.44
Slope	-2.29	0.47	-3.45	-1.13
t-value	2.08			
p-value	0.092			

Sensitivity Analysis for Pre-Post Correlation Assumption in Single-Arm Studies

To assess whether the assumed correlation (r = 0.5) influenced the pooled effect estimate for the five single-arm studies, a sensitivity analysis was performed using alternative correlation values of r = 0.3 and r = 0.7. The pooled standardized mean change for the four single-arm studies with calculable PSS data remained identical (SMD = -1.02) across all assumptions, as the mean difference and pooled SD were unaffected by the correlation assumption. However, the 95% CI varied as follows: for r = 0.3, the CI was -1.38 to -0.66 (wider, reflecting greater uncertainty); for r = 0.5 (primary analysis), the CI was -1.30 to -0.74; and for r = 0.7, the CI was -1.22 to -0.82 (narrower, reflecting higher assumed precision). The primary analysis with r = 0.5 was retained as a conservative estimate, and the overall conclusion of a beneficial association of pranayama with stress reduction remained robust across all assumed correlation values.

*Meta*-*Analysis* *Findings*

**Figure 5:**
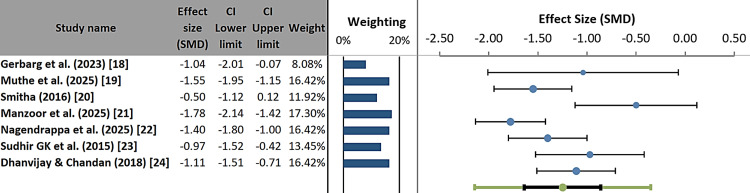
Forest plot of the random-effects meta-analysis showing the effect of pranayama interventions on stress and anxiety in medical students, with pooled estimate and heterogeneity statistics CI: confidence interval, SMD: standardized mean difference

Forest plot and heterogeneity assessment: The total effect size was large and significant (SMD = -1.25, -1.64 to -0.86, p < 0.001), indicating a substantial decrease in stress/anxiety in favor of the yoga breathing groups. Nevertheless, substantial statistical heterogeneity was observed (I² = 67%, p = 0.006), indicating variation in true effect sizes across studies and sampling error (Figure 5) [25].

*Subgroup* *Analysis*

**Figure 6:**
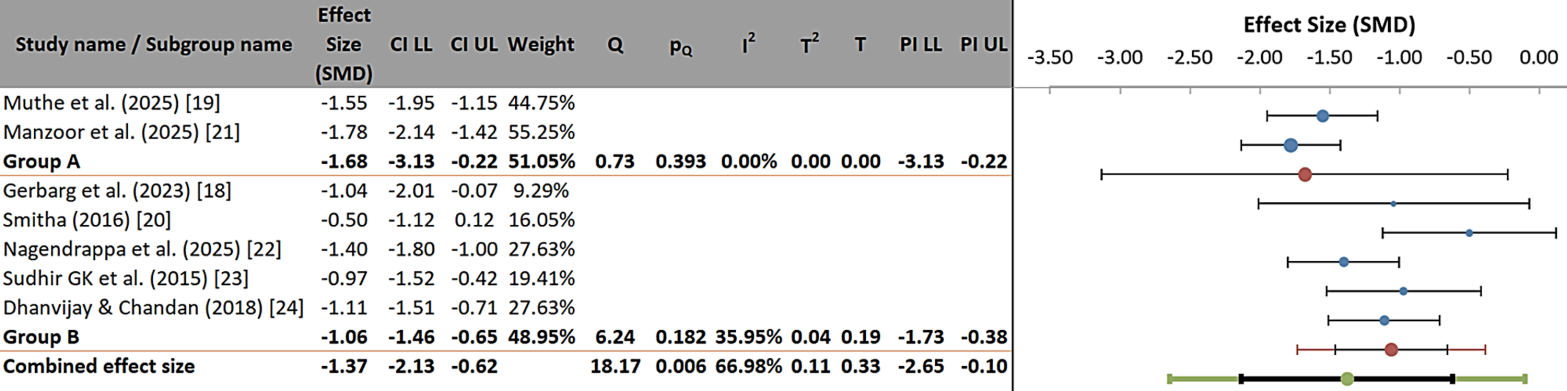
Subgroup analysis forest plot comparing short-term (≤8 weeks) vs. long-term (>8 weeks) pranayama interventions on stress and anxiety in medical students CI: confidence interval, PI: prediction interval, SMD: standardized mean difference, LL: lower limit, UL: upper limit

The short-term intervention (≤8 weeks; Group A) and long-term intervention (>8 weeks; Group B) effect sizes were very large, with SMDs of -1.68 and -1.06, respectively (95% CIs: -3.13 to -0.22 and -1.46 to -0.65). The statistical test of subgroup differences (ANOVA between groups, Q = 9.71, p = 0.002) was also significant, and the effect sizes differed by duration, with short-term interventions possibly yielding a larger pooled effect. The fact that, in the long-term group, the residual heterogeneity was high (I² = 35.95, p = 0.182) indicates that other specific conditions (e.g., technique used, supervision) contribute to variation in outcomes in longer programs. The overall effect in combination is large and significant (SMD = -1.37, p = 0.026), and the overall beneficial effect of pranayama is significant across durations, possibly with a potential trend, where shorter, more intensive programs might be the most effective, as more comparative research is required (Figure 6).

**Figure 7:**
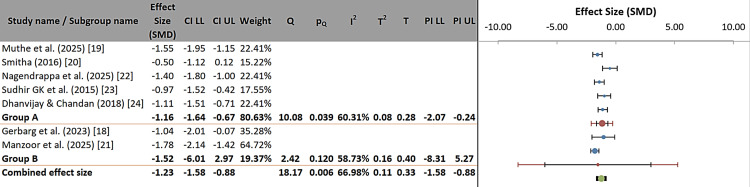
Subgroup analysis forest plot comparing pranayama intervention effects measured using the PSS vs. other scales in medical students CI: confidence interval, PI: prediction interval, SMD: standardized mean difference, LL: lower limit, UL: upper limit, PSS: perceived stress scale

The effect size of the studies that applied PSS (Group A) was also large and statistically significant (SMD = -1.16, 95% CI: -1.64 to -0.67). Conversely, the pooled effect among studies using other scales (Group B), which involved measures of depression (PHQ-9) and general stress/anxiety (DASS-21), was also large but with an extremely broad and non-significant CI (SMD = -1.52, 95% CI: -6.01 to 2.97), due mainly to the small number of studies (n = 2) and also the high level of heterogeneity in this subgroup. The formal test for subgroup differences was not significant (between-groups Q = 0.87, p = 0.352), indicating no statistically significant evidence that the selection of the outcome scale (PSS vs. others) had a systematic effect on the estimated effect size. The summed effect of all scales was large and significant (SMD = -1.23, p < 0.001). It implies that the positive impact of pranayama on psychological distress is stronger than that reported by various validated measurement scales and that the PSS subgroup yields a more accurate and valid estimate, as more studies use it (Figure 7).

**Figure 8:**
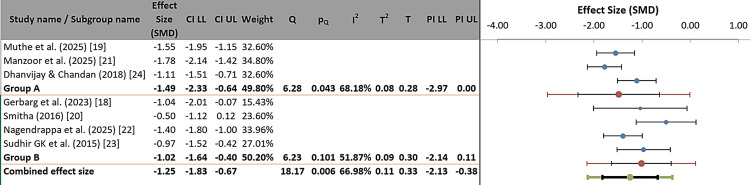
Subgroup analysis forest plot comparing the effect of pranayama interventions by delivery mode (structured and supervised vs. minimally supervised) in medical students CI: confidence interval, PI: prediction interval, SMD: standardized mean difference, LL: lower limit, UL: upper limit, PSS: perceived stress scale

The effect size for structured, supervised programs (Group A) delivered by qualified instructors with frequent supervision was enormous (SMD = -1.49, 95% CI: -2.33 to -0.64). The effect of initial guidance (Group B), with the primary focus on the independent practice, was also significant (SMD = -1.02, -1.64 to -0.40). The subgroup difference test was not significant (between-groups Q = 2.69, p = 0.101), indicating insufficient evidence to conclude that one mode of delivery is better or worse than the other. The heterogeneity in both subgroups was moderate to high (I² = 68.18% and 51.87%, respectively). The overall pooled effect was also very large (SMD = -1.25, p < 0.001), demonstrating the effectiveness of pranayama across all delivery forms (Figure 8).

Discussion

The findings of the current systematic review and meta-analysis demonstrated that pranayama interventions were associated with a statistically significant combined effect on stress and anxiety levels among medical students worldwide [3]. The random-effects model demonstrated a significant pooled effect size (SMD = -1.25). This finding was in line with the broader body of research on mind-body interventions to support student mental health, which has often reported moderate-to-large effects of practices such as mindfulness meditation [18].

Yet, the effect size was significantly bigger in this review than is common with generic mindfulness programs, possibly pointing to the uniquely powerful effect of direct autonomic regulation through breath control, which is an example of mechanistic action that has been backed by prior studies of the physiology [5,6,8]. The high heterogeneity (I² = 67%) was not surprising, as the intervention protocols, timeframes, and outcome measures varied across the studies.

Further details were provided through subgroup analyses. The intervention duration showed a significant difference with the very large pooled effect of short-term programs (≤8 weeks) (SMD = -1.68) and the large effect of long-term programs (SMD = -1.06). This tendency suggested that even brief, systematic pranayama training might have a significant psychological impact, an encouraging development for time-restricted medical programs. This was consistent with other short-term intervention studies in academic institutions where intensive practices tend to yield quick improvements in well-being [4,5].

Conversely, the subgroup analyses based on the primary outcome measure (PSS vs. other scales) and the type of delivery (structured vs. minimally supervised) did not show any statistically significant differences, which reflected that the beneficial effect was not contingent on the measurement tool or implementation format [18-24]. The insignificant delivery-mode result was also encouraging, as it suggested that pranayama might be effective even with limited supervision, which would likely increase its use on a wide scale.

In interpreting these findings, the key risk of bias in the majority of the included studies (mostly uncontrolled pre-post designs) warranted caution [14]. The observed effects may have been inflated by placebo, expectation, or natural temporal changes, which could make them appear stronger. Nevertheless, the quality of the single RCT was high. The risk of bias was low, which in turn indicated a very large effect size (SMD = -1.78) relative to an active physical exercise control, providing the research with significant credibility for the causal conclusion that pranayama practice can reduce distress [21].

This result was well aligned with previous literature investigating the use of physical exercise alone, which, in many cases, has more modest effects on psychological stress among student populations [1,2]. The meta-analysis, therefore, added to the evidence base by isolating the breath component of yoga, indicating that pranayama, in its own right, can be a potent, effective, and affordable intervention to reduce stress prevalent in medical training.

The methodological weaknesses of the included studies substantially constrained the interpretation of these findings. Six of the seven included studies were uncontrolled, single-arm pre-post designs, which posed a critical risk of bias due to confounding by time trends, regression to the mean, placebo effects, and the natural history of stress symptoms [14]. The strong effects observed in these studies could be attributable, in whole or in part, to these factors rather than to the pranayama intervention itself [18, 20, 22-24]. While the single RCT provided higher-quality evidence supporting a causal effect, it accounted for only a portion of the total sample weight in the primary analysis [21]. The meta-analysis results were therefore disproportionately influenced by lower-quality evidence.

Furthermore, the substantial statistical heterogeneity (I² = 67%, p = 0.006) indicated that the true effect sizes varied considerably across studies. While subgroup and sensitivity analyses were conducted to explore this heterogeneity, the small number of studies limited the ability to identify all sources of variation. Residual confounding from unmeasured variables (e.g., baseline stress severity, concurrent interventions, adherence rates, and motivation levels) likely contributed to the observed heterogeneity.

The assessment of publication bias was statistically underpowered because formal tests of funnel plot asymmetry are not recommended when fewer than 10 studies are included. With only seven studies, the non-significant Egger's test (p = 0.092) could not definitively rule out publication bias [15]. It remained possible that smaller studies with null or negative results remained unpublished, potentially inflating the pooled effect estimate. Additionally, a substantial number of full-text reports could not be retrieved despite systematic attempts, potentially introducing retrieval bias. The majority of these were conference abstracts or dissertations, which may have contained data that would have altered the findings [10].

Despite these limitations, several strengths of this review were acknowledged. The comprehensive search strategy across seven databases with manual reference checking minimized the risk of missing relevant studies. The rigorous application of PRISMA guidelines and dual-independent screening, data extraction, and risk-of-bias assessment enhanced reproducibility and reduced reviewer bias [10]. The use of both the RoB 2 and ROBINS-I tools provided a nuanced understanding of the methodological quality of the evidence base [13,14]. The sensitivity analyses excluding uncontrolled studies and varying correlation assumptions demonstrated the robustness of the primary findings to key analytical decisions.

Taking a balanced view of the evidence, pranayama interventions appeared to be a promising, low-cost, and feasible approach for reducing stress and anxiety in medical students. The finding that significant effects were observed even in short-term programs (five to six weeks) was particularly relevant for time-constrained medical curricula [19, 21]. However, given the methodological weaknesses of the evidence base, these findings should be considered hypothesis-generating rather than conclusive. Educators and administrators considering implementing pranayama programs should interpret effect sizes with caution and prioritize rigorous program evaluation. The non-significant difference between structured and minimally supervised delivery modes suggested that lower-intensity, lower-resource approaches might still confer benefits, enhancing scalability.

Study limitations

Several limitations were acknowledged in this review. The most significant constraint was the predominance of studies with a high risk of bias, as six of the seven included studies were single-arm pre-post designs assessed as having a serious or critical risk of bias using the ROBINS-I tool [14]. A further limitation concerned the analysis of single-arm studies, which required assuming a pre-post correlation (r = 0.5) for variance calculation, as the original authors did not report these correlations. While sensitivity analyses using alternative correlations (r = 0.3 and r = 0.7) confirmed the robustness of the pooled effect size, the precision of the CIs was influenced by the assumption underlying these correlations. A notable limitation was the limited geographic diversity of the included studies. Six of the seven studies (85.7%) were conducted in India [19-24], with one in Puerto Rico/USA [18]. No studies from other global regions, such as Europe, East Asia, Africa, or Australia, met the inclusion criteria. This geographic concentration limited the generalizability of the findings to medical student populations in other cultural and educational contexts, where stress perceptions, coping mechanisms, and responses to mind-body interventions may differ substantially.

Additionally, the assessment of publication bias was underpowered because formal tests of funnel plot asymmetry are not recommended for fewer than 10 studies. With only seven studies, the non-significant Egger's test (p = 0.092) could not definitively rule out publication bias [15]. The search was restricted to English-language publications, which could have introduced language bias and limited global representativeness. Furthermore, 39 full-text reports could not be retrieved despite systematic attempts, potentially introducing retrieval bias. The majority of these were conference abstracts or dissertations, which may have contained data that would have altered the findings. The significant statistical heterogeneity (I² = 67%) indicated that unmeasured variables influenced the outcomes, despite efforts to explore this through subgroup and sensitivity analyses. Finally, the relatively small number of included studies (n = 7) limited the statistical power of subgroup analyses. It precluded the use of more advanced meta-regression techniques to explore sources of heterogeneity.

Future directions

Future studies ought to focus on rigorously structured RCTs with active control groups to conclusively demonstrate that pranayama is an effective practice. Comparisons of various pranayama methods (e.g., HFYB vs. LFYB) and time periods should be conducted through studies to determine the best protocols. A critical priority is the standardization of intervention protocols, including specifying the exact sequence and duration of each pranayama technique, frequency and intensity of practice, instructor qualifications, and procedures for monitoring adherence, as the substantial variability observed across included studies in techniques, session durations, and total periods limited comparability and synthesis. Similarly, standardization of outcome measures is urgently needed; future research should prioritize the use of widely accepted, validated instruments such as the PSS-10 and STAI-S to facilitate meta-analytic synthesis and reduce statistical heterogeneity. The biological basis of these interventions, including alterations in HRV, cortisol, and neural correlates, could be better understood by investigating the underlying biological processes. Longitudinal research is required to establish how the benefits can be sustained and integrated into medical students' long-term lifestyles. Additionally, more studies are needed on implementation science strategies for integrating viable, scalable pranayama programs across medical schools worldwide. Finally, future studies should adhere to reporting guidelines such as CONSORT, with complete reporting of means, SDs, and pre-post correlations to enable accurate meta-analysis.

## Conclusions

This systematic review and meta-analysis found that pranayama interventions were associated with statistically significant reductions in perceived stress and anxiety among medical students in the included studies. However, because most of the evidence came from uncontrolled studies with a high risk of bias, causal conclusions cannot be drawn. The only RCT available provided preliminary support for efficacy, but replication is needed. While pranayama is a promising, low-cost, and feasible intervention for student wellness programs, its effectiveness has not yet been definitively established. Future high-quality RCTs are required before strong recommendations can be made.
